# Effectiveness and safety of outpatient rehabilitation versus home-based rehabilitation after knee arthroplasty: a systematic review and meta-analysis

**DOI:** 10.1186/s13018-023-04160-2

**Published:** 2023-09-19

**Authors:** BiXia Zhao, Hui Liu, Ke Du, Wei Zhou, Ying Li

**Affiliations:** 1 Department of Nursing, The Affiliated Nanhua Hospital, Hengyang Medical School, University of South China, 28 Changsheng West Road, Hengyang, 421001 Hunan Province China; 2 Department of Supervision Office, The Affiliated Nanhua Hospital, Hengyang Medical School, University of South China, 28 Changsheng West Road, Hengyang, 421001 Hunan Province China; 3 Department of Emergency, The Affiliated Nanhua Hospital, Hengyang Medical School, University of South China, 28 Changsheng West Road, Hengyang, 421001 Hunan Province China; 4 Department of Hand and Foot Surgery, The Affiliated Nanhua Hospital, Hengyang Medical School, University of South China, 28 Changsheng West Road, Hengyang, 421001 Hunan Province China

**Keywords:** Knee arthroplasty, Rehabilitation, Outpatient, Home-based, Meta-analysis

## Abstract

**Background:**

Rehabilitation post-knee arthroplasty is integral to regaining knee function and ensuring patients’ overall well-being. The debate over the relative effectiveness and safety of outpatient versus home-based rehabilitation persists.

**Methods:**

A thorough literature review was conducted adhering to Preferred Reporting Items for Systematic Reviews and Meta-Analyses (PRISMA) guidelines across four databases. Two researchers independently identified eligible studies centering on knee arthroplasty patients undergoing either outpatient or home-based rehabilitation. Study quality was assessed using the Cochrane Collaboration’s risk of bias tool, while continuous outcomes were subject to meta-analyses using Stata 17 software.

**Results:**

Our analysis identified no significant differences in primary outcomes, including Range of Motion, Western Ontario and McMaster Universities Arthritis Index, Knee Injury and Osteoarthritis Outcome Score, Oxford Knee Score, and the Knee Society Score, between home-based and outpatient rehabilitation across different follow-up points. Adverse reactions, readmission rates, the need for manipulation under anesthesia, reoperation rate, and post-surgery complications were also similar between both groups. Home-based rehabilitation demonstrated cost-effectiveness, resulting in substantial annual savings. Furthermore, quality of life and patient satisfaction were found to be comparable in both rehabilitation methods.

**Conclusions:**

Home-based rehabilitation post-knee arthroplasty appears as an effective, safe, and cost-efficient alternative to outpatient rehabilitation. Despite these findings, further multicenter, long-term randomized controlled trials are required to validate these findings and provide robust evidence to inform early rehabilitation choices post-knee arthroplasty.

## Introduction

Knee arthroplasty is a renowned surgical procedure for end-stage osteoarthritis (OA) that has proven its worth over time [1]. With global demographic shifts toward aging populations and escalating obesity rates, the demand for knee arthroplasty surgeries has seen a precipitous surge. Nonetheless, it is crucial to understand that successful outcomes from knee arthroplasty extend beyond the surgical intervention itself and rely heavily on postoperative rehabilitation [2, 3].

Postoperative rehabilitation plays a pivotal role in fostering adherence to the therapeutic regimen, expediting the recovery of knee function, and heightening patient satisfaction [4]. However, there exist significant disparities in the early postoperative rehabilitation modalities implemented across various medical institutions worldwide. Outpatient rehabilitation is one approach favored by many healthcare providers due to its high professionalism and safety. It typically involves regular visits to a healthcare facility, where patients receive treatment from trained professionals. This approach, while robust, can pose certain challenges related to cost, time, and accessibility. These aspects may potentially impair adherence and yield less favorable outcomes, especially among the elderly and those residing in remote geographical locations. In contrast, home-based rehabilitation has gained substantial attention as an alternative approach [5, 6]. This method of rehabilitation offers the comfort of familiar surroundings and potential flexibility in scheduling, which can enhance the patient’s motivation and compliance with the therapy regimen. Reduced travel time and expenses also constitute significant advantages. However, despite the apparent benefits, the safety and efficacy of home-based rehabilitation in relation to outpatient rehabilitation following knee arthroplasty remain largely unexplored and substantiated in the medical literature.

This knowledge gap necessitates a comprehensive investigation into the relative effectiveness and safety of these two prevalent rehabilitation paradigms. Accordingly, our study conducted a systematic review and meta-analysis to delve into this issue. Our goal is to equip healthcare providers, patients, and policymakers with evidence-based insights to facilitate informed decision-making in selecting the optimal early postoperative rehabilitation approach after knee arthroplasty. In the broader context, our findings will contribute to the ongoing discourse on enhancing patient-centric care. By aligning rehabilitation strategies with individual patient preferences, circumstances, and needs, we can ensure more tailored and effective healthcare solutions. These endeavors ultimately aimed to optimize the overall outcomes of knee arthroplasty, significantly improving the quality of life for those affected by end-stage OA.

## Materials and methods

During the systematic review process and subsequent reporting of our results, we maintained adherence to the Preferred Reporting Items for Systematic Reviews and Meta-Analyses (PRISMA) guidelines [7]. Since the information utilized in this article was sourced from published materials, there was no need for informed consent or ethical approval. Two researchers conducted a systematic search of pertinent studies, independently determined their eligibility, extracted data, and evaluated the quality of the research. The two researchers were required to reach a consensus and resolve any points of disagreement. During the systematic review process, two researchers, BZ and KD, independently conducted the article screening, data extraction, and risk of bias assessment. Any disagreements were resolved through consensus. To keep track and manage the process of article screening and data extraction, we utilized the Covidence software tool.

### Search strategy

Four electronic databases PubMed, Embase, Web of Science, and Cochrane Library were searched on May 6, 2023 and no time limitation was applied. Vocabulary and syntax were specifically adapted according to the database. The specific search terms of PubMed were: ((“Arthroplasty” [Title/Abstract] OR “Arthroplasty, Replacement, Knee” [MeSH Terms]) AND (“Rehabilitation” [Title/Abstract] OR “Rehabilitation” [MeSH Terms]) AND ((“Outpatient” [Title/Abstract] OR “Ambulatory Care” [MeSH Terms]) OR (“Home-Based” [Title/Abstract] OR “Home Care Services” [MeSH Terms])). No language limitation was applied. Reference lists of relevant articles were also screened manually for any additional possible records.

### Inclusion criteria and exclusion criteria

Studies included in the systematic review needed to meet the following criteria: (1) The subjects of the study must be patients who have undergone knee arthroplasty; (2) The intervention measures should be: the experimental group undergoes outpatient rehabilitation, while the control group undergoes home-based rehabilitation; (3) Outcome indicators should include range of motion (ROM), Western Ontario and McMaster Universities Arthritis Index (WOMAC), Knee Injury and Osteoarthritis Outcome Score (KOOS), Oxford Knee Score (OKS), and Knee Society Score (KSS); (4) If a study involving the same population is published more than once, select the study with the largest sample size or the newly published study. The subjects of the study must be patients who have undergone total knee arthroplasty. The study did not include patients who had undergone unicompartmental, bilateral, or revision arthroplasty procedures.

The exclusion criteria were as follows: (1) Studies that are duplicates or redundant publications; (2) Studies that do not include the required outcome indicators; (3) Studies with data that is unable to be extracted or with incomplete literature; (4) Case reports, commentaries, expert opinion, and narrative reviews.

### Data extraction

Data extraction was carried out independently by two systematically trained researchers based on the inclusion and exclusion criteria. The information extracted from the literature included basic information (author, age, gender), intervention measures, outcome indicators, and measurement time points for each study. When there was no data of interest in the published report, we contacted the investigators of the original study by email to request the unpublished data.

### Quality assessment

The quality of the included studies was assessed by the Cochrane Collaboration’s risk of bias tool [8]. Two reviewers (BZ and KD) independently evaluated the following domains: random sequence generation, allocation concealment, blinding of participants and personnel, incomplete outcome data, selective reporting, and other potential sources of bias. Each domain was judged as having a low, unclear, or high risk of bias. Disagreements between reviewers were resolved through discussion or consultation with a third reviewer, if necessary.

### Statistical analyses

Meta-analyses were conducted using Stata version 17 (StataCorp, College Station, TX, USA). For this study, all the outcome indicators to be combined are continuous variables. As the outcome indicators are measured using the same method and identical units, results will be represented by the mean difference (MD) and 95% confidence intervals (CI). Firstly, heterogeneity tests were performed on the study results. If there is no significant statistical heterogeneity between the studies (*P* > 0.05, *I*^2^ < 50%), a fixed-effects model will be used for the analysis. If statistical heterogeneity was present (*P* ≤ 0.05, *I*^2^ ≥ 50%), a random-effects model will be applied to combine the effect sizes. If the heterogeneity is too obvious and its source is indeterminable, a descriptive analysis will be carried out instead. Differences will be considered statistically significant at *P* < 0.05.

## Results

### Search results and study selection

**Fig. 1:**
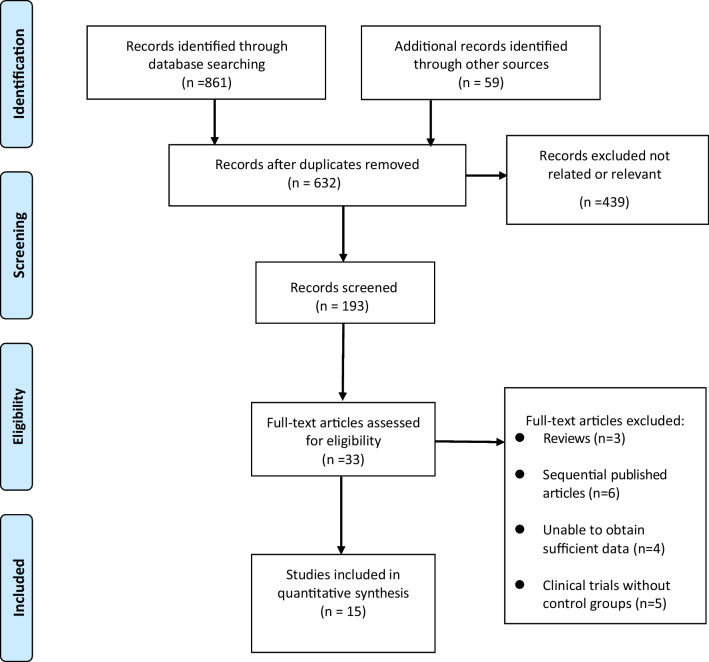
Selection process of included studies

From the initial search of the electronic databases, 920 related studies were initially found. After removing repetitive studies, reading titles and abstracts, and screening strictly according to the inclusion and exclusion criteria, 33 related studies were obtained, and 18 were excluded from further reading. Finally, 15 articles were included [9–23]. The literature screening process and results are shown in Fig. 1.

### Study characteristics

The characteristics of studies included in this systematic review are presented in Table 1. This meta-analysis included fifteen studies, with patients’ ages ranging from approximately 60 to 70 years in both the outpatient and home-based rehabilitation groups. The female-to-male ratios varied widely across studies. The interventions differed in terms of their frequency and duration between outpatient and home-based settings. The outcome measures evaluated in these studies were diverse and included the range of motion (ROM), Western Ontario and McMaster Universities Arthritis Index (WOMAC), Knee Injury and Osteoarthritis Outcome Score (KOOS), Oxford Knee Score (OKS), and the Knee Society Score (KSS). Most studies assessed multiple outcomes, but the specific outcomes evaluated varied from study to study. In terms of follow-up durations, studies employed a variety of time points, with many evaluating outcomes at multiple time points ranging from 1.5 to 24 months post-intervention.

**Table 1:** Characteristics of studies included in the meta-analysis

Included studies	Age (Years, x or x ± s) outpatient group/home group	Gender ratio (female/male) outpatient group/home group	Outcome measures	Measurement time point (months)	Intervention measures—experimental group	Intervention measures—control group
Fleischman et al. [13]	65/66	47/3: 48/1	Range of Motion, Knee Injury and Osteoarthritis Outcome Score	6	Outpatient rehabilitation + supplementary home exercise, 2–3 times/week for 4–8 weeks	Home-based rehabilitation, 3 times/week for 8 weeks
Fillingham et al. [12]	60 ± 7.4/60 ± 7.8	16/9: 13/9	Western Ontario and McMaster Universities Arthritis Index, Knee Society Score	1.5	Outpatient rehabilitation, 3 times/week for 6 weeks	Home-based rehabilitation daily for 30–45 min/session, intensity gradually increases over 6 weeks
Barker et al. [10]	70.18 ± 8.14/70.67 ± 8.01	187/125: 184/125	Knee Injury and Osteoarthritis Outcome Score, Oxford Knee Score	12	Outpatient rehabilitation + supplementary home exercise, 1–6 times	Home-based rehabilitation
Hamilton et al. [14]	66.80 ± 9.46/68.20 ± 9.44	97/66: 108/63	Oxford Knee Score	12	Outpatient rehabilitation + supplementary home exercise, 2 times/week for 6 weeks	Home-based rehabilitation, 3 times/week for 6 weeks
Büker et al. [11]	64.25 ± 3.86/68.08 ± 6.25	16/2: 15/1	Range of Motion, Western Ontario and McMaster Universities Arthritis Index	24	Outpatient rehabilitation, 5 days/week for 4 weeks	Home-based rehabilitation, 1 h/day, 5 days/week for 4 weeks
Han et al. [15]	65.40 ± 6.0/64.1 ± 6.5	104/92: 108/86	Western Ontario and McMaster Universities Arthritis Index	1.5	Outpatient rehabilitation for 6 weeks	Home-based rehabilitation, 10 times/session, 3 times/day for 6 weeks
Ko et al. [17]	N/A	58/27: 44/36	Range of Motion, Western Ontario and McMaster Universities Arthritis Index	12	Outpatient and home-based rehabilitation, 2 times/week for 6 weeks	Home-based rehabilitation, 3 times/day for 12 weeks; thereafter, once/day indefinitely
Kramer et al. [18]	68.2 ± 6.9/68.6 ± 7.8	44/36: 47/33	Range of Motion, Western Ontario and McMaster Universities Arthritis Index	12	Outpatient rehabilitation + supplementary home exercise, 1 h/session, 1–2 times/week for 12 weeks; may continue beyond 12 weeks based on doctor’s recommendation	Home-based rehabilitation
Levine et al. [19]	65.1/68.1	21/14: 25/10	Range of Motion, Western Ontario and McMaster Universities Arthritis Index	6	Outpatient rehabilitation + supplementary home exercise, 60 days	Home-based rehabilitation, 6 weeks duration
Mockford et al. [21]	69.4/70.9	46/25: 42/30	Knee Society Score	12	Outpatient rehabilitation + supplementary home exercise, 6 weeks duration	Home-based rehabilitation, 6 weeks duration
Rajan et al. [22]	69.0 ± 9.3/68.0 ± 10.0	36/20: 37/23	Range of Motion, Oxford Knee Score	12	Outpatient rehabilitation + supplementary home exercise, average 4–6 times	Home-based rehabilitation, started pre-operation
Kauppila et al. [16]	70.7 ± 5.7/70.6 ± 5.3	32/12: 33/9	Range of Motion	12	Outpatient rehabilitation + supplementary home exercise, 10 days duration	Home-based rehabilitation, 20 min/day, at least 5 days/week for 7 weeks; or 2–3 days/week for at least 10 months
Xu et al. [23]	67.3 ± 6.9/68.4 ± 8.4	43/8: 44/11	Western Ontario and McMaster Universities Arthritis Index, Knee Society Score	12	Outpatient rehabilitation, first 7 weeks, 2 days/week, then 1 day/week for the following 10 months, total 24 times	Home-based rehabilitation, 2 times/week, 6 weeks duration
Madsen et al. [20]	66.9 ± 8.5/66.2 ± 8.2	17/23: 16/24	Western Ontario and McMaster Universities Arthritis Index, Knee Society Score	6	Outpatient rehabilitation + supplementary home exercise, 2 times/week, 6 weeks duration	Home-based rehabilitation
Artz et al. [9]	70.0/67.2	12/11: 12/11	Knee Injury and Osteoarthritis Outcome Score	6	Outpatient rehabilitation + supplementary home exercise, 6 weeks duration	Home-based rehabilitation, 3 times/day, indefinite continuation after 12 weeks

### Results of quality assessment

**Fig. 2:**
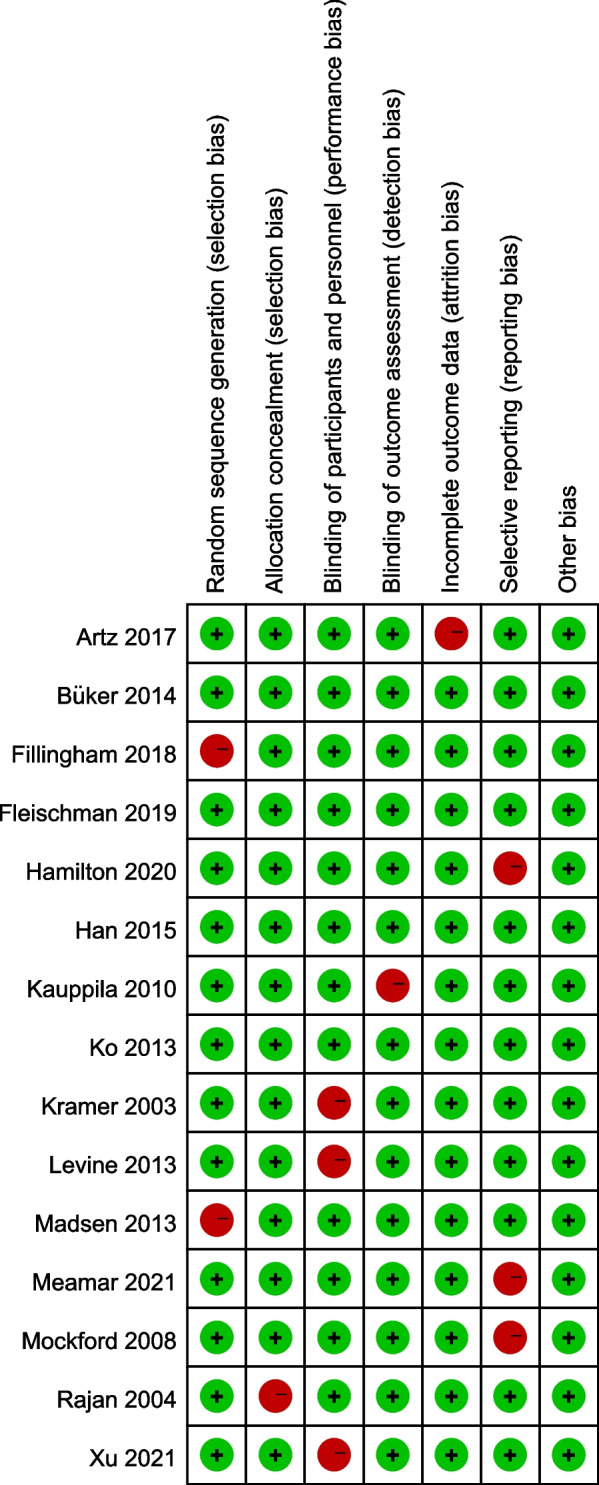
Quality assessment of included studies using Cochrane Collaboration’s risk of bias tool

The evaluation of bias risk was conducted across multiple domains in the 15 studies that were included. Seven studies demonstrated a low risk of bias in all categories, indicating a high level of methodological rigor. However, upon further examination, we found that the domain of blinding of participants and personnel might require a more nuanced view. Specifically, we noted that 20% of the studies were found to have a high risk of performance bias due to patients not being blinded, which is inherent in many surgical or physiotherapy interventions. This risk is particularly pronounced for patient-reported outcome measures (PROMs) such as WOMAC and KOOS, where participants, aware of their group assignments, were their own assessors. This potential for performance bias might have influenced the outcomes of these studies, and hence, the interpretation of the results needs to take this factor into account. For objective measures like ROM, blinding of the outcome assessment is essential and more feasible. For such measures, the risk of performance bias is significantly reduced when the assessor is unaware of the participant group assignments. Moreover, in 21% of the included randomized controlled trials, a high risk of selective reporting bias was observed. This indicated that the possibility of incomplete or selective outcome reporting might have affected the overall results of these studies (Fig. 2).

### Range of joint motion

**Fig. 3:**
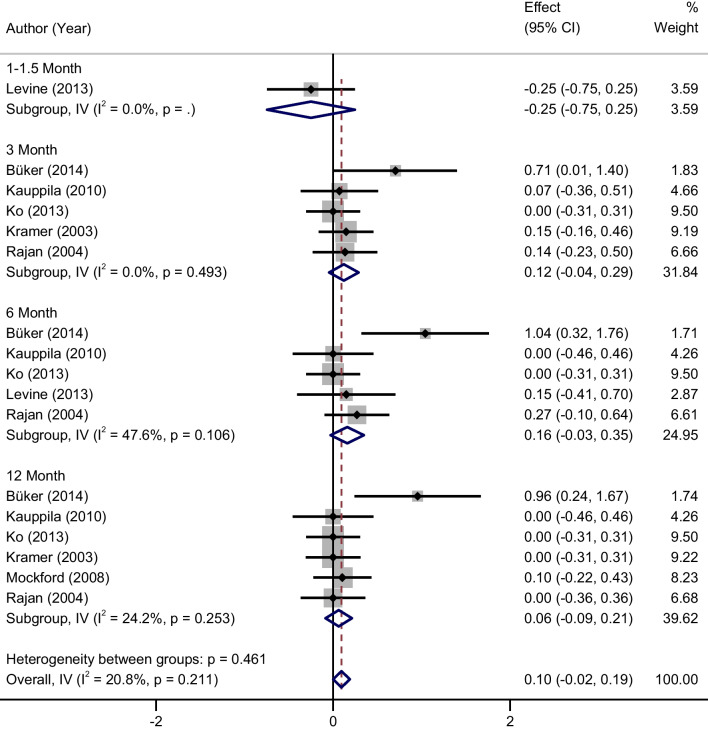
Forest plots of the changes in knee flexion between two groups

Variation in knee joint flexion. There was no significant heterogeneity between studies (*P* = 0.211, *I*^2^ = 33%), and a fixed effect model was used for meta-analysis. There were no statistically significant differences in the changes in knee flexion between the home rehabilitation group and the outpatient rehabilitation group at 1–1.5, 3, 6, 12 months post-surgery (1–1.5 months: MD = − 0.25, 95% CI (− 0.75, 0.25); 3 months: MD = 0.12, 95% CI (− 0.04, 0.29); 6 months: MD = 0.16, 95% CI (− 0.03, 0.35); 12 months: MD = 0.06, 95% CI (− 0.09, 0.21) (Fig. 3).

**Fig. 4:**
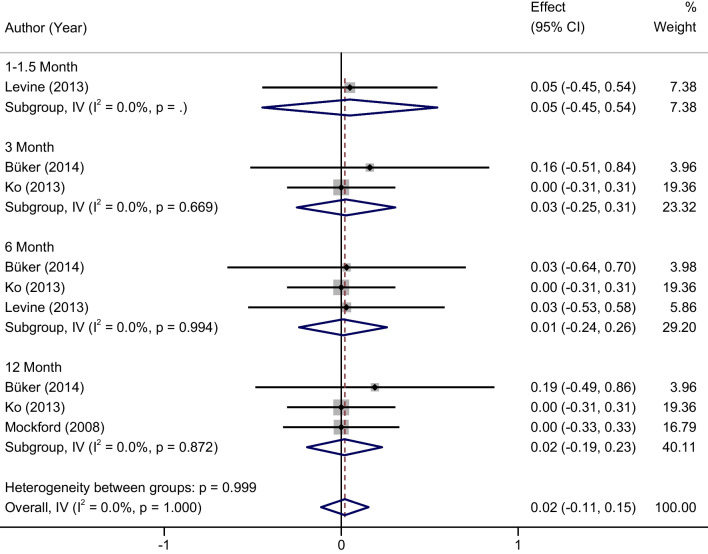
Forest plots of the changes in knee extension between two groups

Variation in knee joint extension. No significant heterogeneity was found between studies (*P* = 1.00, *I*^2^ = 0%), so a fixed effect model was used for meta-analysis. There were no statistically significant differences in the changes in knee extension between the home rehabilitation group and the outpatient rehabilitation group at 1–1.5, 3, 6, 12 months post-surgery (1–1.5 months: MD = 0.05, 95% CI (− 0.45, 0.54); 3 months: MD = 0.03, 95% CI (− 0.25, 0.31); 6 months: MD = 0.01, 95% CI (− 0.24, 0.26); 12 months: MD = 0.02, 95% CI (− 0.19, 0.23) (Fig. 4).

### Joint injury and osteoarthritic results

Variation in osteoarthritis index score. Six studies [11, 15, 17–19, 23] reported on the change in WOMAC (Western Ontario and McMaster Universities Osteoarthritis Index) post-knee replacement surgery. There was significant heterogeneity between studies (*P* < 0.05, *I*^2^ = 61%), so a random effect model was used for meta-analysis. There were no statistically significant differences in the changes in WOMAC between the home rehabilitation group and the outpatient rehabilitation group at 1–1.5, 3, 6, 12 months post-surgery (1–1.5 months: MD = 1.56, 95% CI (− 1.65, 5.61); 3 months: MD = − 2.68, 95% CI (− 6.31, 0.16); 6 months: MD = − 0.26, 95% CI (− 3.56, 3.61); 12 months: MD = 1.56, 95% CI (− 1.63, 3.67).

Variation in knee injury and osteoarthritis outcome score. Four studies [9, 10, 12, 13] reported on the change in KOOS (Knee injury and Osteoarthritis Outcome Score) post-knee replacement surgery. No significant heterogeneity was found between studies (*P* = 0.65, *I*^2^ = 0%), so a fixed effect model was used for meta-analysis. There were no statistically significant differences in the changes in KOOS between the home rehabilitation group and the outpatient rehabilitation group at 1–1.5, 3, 6, 12 months post-surgery (1–1.5 months: MD = − 1.65, 95% CI (− 3.16, 2.35)]; 3 months: MD = 5.61, 95% CI (− 2.65, 9.36); 6 months: MD = − 0.16, 95% CI (− 2.16, 3.65); 12 months: MD = 0.31, 95% CI (− 3.65, 2.65).

### Knee function score

Variation in Oxford knee score. Four studies [10, 14, 20, 21] reported on the change in OKS (Oxford Knee Score) post-knee replacement surgery. No significant heterogeneity was found between studies (*P* = 0.16, *I*^2^ = 36%), so a fixed effect model was used for meta-analysis. There were no statistically significant differences in the changes in OKS between the home rehabilitation group and the outpatient rehabilitation group at 3, 6, 12 months post-surgery (3 months: MD = 0.56, 95% CI (− 0.81, 1.56)]; 6 months: MD = 0.26, 95% CI (− 0.75, 1.46); 12 months: MD = 0.52, 95% CI (− 0.89, 1.87).

Knee Joint Function Score Variation. Three studies [12, 19, 23] reported the changes in the Knee Society Score (KSS) of patients after knee joint replacement surgery. There was no significant heterogeneity among the studies (*P* = 0.69, *I*^2^ = 0%), and a fixed effect model was adopted for the meta-analysis. No statistically significant differences were found in the changes in KSS between the home rehabilitation group and the outpatient rehabilitation group at 1–1.5, 3-, 6-, and 12-month post-surgery.

### Safety indicators

Five studies [10, 12, 13, 15, 23] reported comparisons of adverse reactions between the two groups. No statistically significant differences were found in terms of readmission rates, need for manipulation under anesthesia, or reoperation rate in any of the studies. Moreover, no significant adverse reactions or complications, including deep vein thrombosis, pulmonary embolism, superficial and deep infections, periprosthetic fractures, or joint stiffness, were reported within six weeks post-surgery in either group.

### Economic indicators

Three studies [10, 11, 23] reported comparisons of the medical costs between two groups. While the measures, parameters, and units varied among the studies, the home rehabilitation group consistently exhibited cost savings compared to the outpatient group. In the study by Buker et al. [11], the total cost for outpatient rehabilitation was reported as 508.6 lira, whereas home rehabilitation was reported to be 299.40 lira over a two-year postoperative period. This implies a saving of approximately 200 lira with home rehabilitation. Barker et al. [10] conducted an economic health analysis and reported an annual saving of 342 pounds for home rehabilitation patients compared to those in outpatient rehabilitation. The overall social cost for home rehabilitation was found to be lower than outpatient rehabilitation (a reduction of 316 pounds per case). From a health and social care perspective, home rehabilitation might present a 43% cost-effectiveness advantage at the same level of utility. Lastly, the study by Xu et al. [23] reported the total rehabilitation cost over the first two months post-discharge to be 1805 yuan for the outpatient group, compared to 1023 yuan for the home rehabilitation group. This suggests that home rehabilitation can save about 800 yuan within the first two months post-discharge. The variance in currencies (Lira, Pounds, Yuan) reflects the diverse geographical locations of these studies. The current rates are approximately 200 lira = 24 USD, 342 pounds = 472 USD, and 800 yuan = 125 USD, but exchange rates can fluctuate. These studies show a common trend of cost savings in home rehabilitation, even though they differ in currencies and healthcare systems.

### Sociological indicators

Seven studies [10–13, 16, 17, 20] compared the quality of life between the two groups, with five studies reporting no significant benefit of outpatient rehabilitation in improving patients’ quality of life. In addition, four studies [9, 14, 17, 19] compared patient satisfaction between the two groups, and all reported no statistically significant differences in satisfaction between patients in the home rehabilitation group and the outpatient rehabilitation group.

### Publication bias

**Fig. 5:**
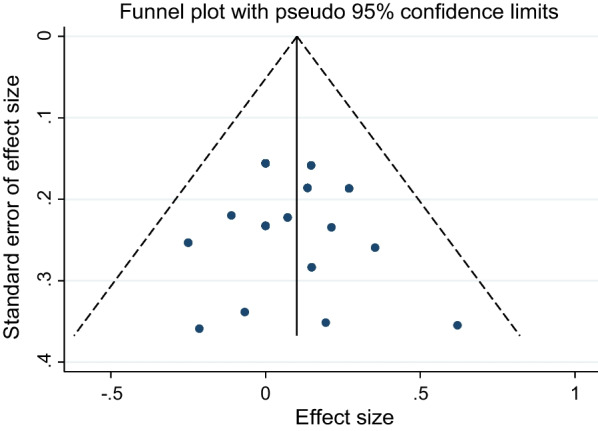
Funnel plot for publication bias in all included studies

The funnel plots constructed with the observed study showed symmetry, and no significant publication bias was detected in funnel plots (Fig. 5).

## Discussion

Our study, through a meticulous systematic review and meta-analysis, reveals that home-based rehabilitation is on par with outpatient rehabilitation in restoring knee function following knee arthroplasty, thereby corroborating several findings reported in the existing literature [10, 11, 14, 15, 17, 24]. Pain, one of the most prevalent postoperative symptoms, is primarily managed by pharmacological interventions, with rehabilitation playing a complementary role. Due to the heterogeneity in pain assessment tools across the included studies and the predominance of drug therapies in pain management, we refrained from conducting a meta-analysis for pain scores. Knee function was primarily evaluated using WOMAC (Western Ontario and McMaster Universities Osteoarthritis Index), KOOS (Knee injury and Osteoarthritis Outcome Score), OKS (Oxford Knee Score), and KSS (Knee Society Score)—commonly used scoring systems in post-arthroplasty scenarios. These scores objectively measure the severity of arthritis symptoms and signs, thus, evaluating the treatment outcome. Importantly, our findings highlighted no significant differences in the changes of ROM (Range of Motion), WOMAC, KOOS, OKS, and KSS scores between home-based and outpatient rehabilitation at various time points (1–1.5, 3-, 6-, and 12-month post-arthroplasty). This suggests that within a year post-arthroplasty, home-based rehabilitation is not inferior to professional outpatient rehabilitation.

Outpatient rehabilitation provides an environment conducive to specialized recovery for patients, particularly those discharged early with conditions like stroke, cardiac diseases, respiratory system diseases, hip fractures, and geriatric ailments. Studies have shown that outpatient rehabilitation can significantly reduce the risks of mortality and adverse prognoses for patients [25]. Although some research suggests that the effectiveness of outpatient rehabilitation is similar to home-based rehabilitation, many patients prefer outpatient settings due to the facilities provided such as gyms and hydrotherapy pools, immediate consultation with professional medical staff, and opportunities for social interaction. TelePT (tele-rehabilitation) has increasingly proven to be a potent tool for delivering physical therapy in a home setting [26]. Therapists can remotely assess and guide patients, considering their home environment and customizing specific rehabilitation plans for them. Moreover, telemedicine reduces the inconvenience of home visits, offering continuous care to patients who cannot easily travel. Monitoring without active treatment, an emerging field, involves the use of smart devices and technologies to remotely observe patients, ensuring they maintain an optimal physical condition without formal treatment [27].

Osteoarthritis (OA) is a primary cause of disability. Total knee arthroplasty (TKA) effectively alleviates pain, corrects deformities, and enhances functionality. Atrophy and voluntary activation (VA) are the primary reasons for quadriceps strength loss, where the impact of VA is nearly double that of muscle atrophy. VA is one of the critical factors for strength loss post-TKA; if a patient’s motor unit cannot achieve its maximal firing rate, a reduction in VA would significantly affect muscle output. However, research on the link between the decline in VA post-TKA and myocardial infarction is still scarce [28]. Several factors that might influence the recovery of the quadriceps muscle after TKA have been studied. While evidence on the efficacy of rehabilitative training remains limited, its safety and positive role should prompt broader research, including its potential feasibility in low-cost models (e.g., group sessions, remote pre-adaptive training) [29]. Mizner et al. [30] demonstrated a significant relationship between quadriceps strength and functional assessments like TUG, stair climbing, 6-min walking, and sit-to-stand tests. Modern rehabilitation integrates conventional functional assessments and specific equipment like leg press, exercise bikes, and biomechanical balance platforms, helping patients recover faster post-TKA. Leg press enhances quadriceps strength; exercise bikes aid in joint mobility recovery and muscle strengthening; while biomechanical balance platforms assess and train balance, providing patients with instant feedback [31, 32]. In conclusion, a combination of various rehabilitation methods is crucial for post-TKA patients, especially when their rehabilitative needs are complex.

At present, both outpatient and home-based rehabilitation play pivotal roles in the field of rehabilitation. Outpatient rehabilitation offers a professional environment, catering to patients with more intricate needs, while home-based rehabilitation provides patients with more convenient and personalized support. For those who require specialized modalities or equipment, such as thermotherapy or the use of short-crank bicycles, home-based rehabilitation might not be entirely adequate. Within an outpatient setting, the provision of professional equipment and techniques ensures that patients receive appropriate treatments. Indeed, numerous studies have indicated that specific equipment and techniques are indispensable in expediting the recovery process [33]. For instance, thermotherapy, as a traditional physical therapy method, has been proven to be exceptionally effective in alleviating pain, enhancing muscle flexibility, and promoting blood circulation [34]. Achieving the therapeutic results provided in clinics might be challenging at home. Similarly, specialized equipment like short-crank bicycles, given their unique design, offers more expert training for particular patient groups, such as those with knee issues, facilitating their recovery [35]. Therefore, although home-based rehabilitation boasts significant advantages like convenience, personalized support, and the potential for remote treatments, outpatient rehabilitation retains irreplaceable value in offering specific equipment and technique-based treatments. The ideal scenario might be an organic combination of home-based and outpatient rehabilitation, ensuring patients receive optimal support and recovery from multiple fronts.

Safety concerns surrounding home-based rehabilitation stem from a lack of professional supervision. However, telemonitoring systems [10, 13, 15] enable therapists to oversee patient progress, enhancing safety. Our analysis suggests that home-based rehabilitation does not compromise patient safety, though more research is needed. This approach offers proven interventions like cryotherapy and mobility techniques right at the patient’s home and has been shown to improve physical activity outcomes post-knee arthroplasty [36].

The rehabilitation journey post-knee arthroplasty is a long-term commitment. Patients’ needs vary based on their educational level, socioeconomic status, and social support system. Outpatient rehabilitation offers specialized care, but its feasibility can be limited for older patients, those with mobility issues, or those with limited access to healthcare resources. Our analysis indicates that home-based rehabilitation is cost-effective, without a compromise on patient quality of life and satisfaction [13], making it a more universally applicable and clinically significant method [11, 23].

However, outcomes can differ based on patient adherence, and the absence of direct supervision might pose risks [12]. Implementing strict protocols, such as telemonitoring, can bolster safety and effectiveness during home-based rehabilitation. Recognizing home-based rehabilitation’s potential, especially post-knee arthroplasty for those with limited healthcare access, the elderly, or mobility-challenged patients is vital. The choice between home-based and outpatient rehabilitation should be individualized, factoring in the patient’s unique conditions and their capacity to follow a regimen without close oversight [10, 14, 23].

In this study, “home-based rehabilitation” encompasses diverse strategies within a home setting, from professional services to unsupervised methods. This broad categorization might introduce clinical heterogeneity affecting our findings. Some reviewed studies reported participants transitioning between or simultaneously initiating home-based and outpatient therapy, which might have impacted treatment distinctions. In future research, we will aim for a more detailed categorization of rehabilitation strategies.

Our study has limitations. We only considered English and Chinese literature, risking potential publication bias. A limited number of studies were included without a bias analysis, increasing clinical heterogeneity. Particularly, ROM measurements in the meta-analysis presented high heterogeneity due to unclear active or passive measurements in primary studies. Additionally, the small sample size for safety indicators suggests the need for more extensive studies for validation.

In conclusion, our systematic review and meta-analysis provide compelling evidence that home-based rehabilitation following knee arthroplasty provides comparable outcomes to outpatient rehabilitation, with satisfactory safety and patient satisfaction, while also offering potential cost savings. This approach does not compromise patient satisfaction, potentially making it more accessible for a broader patient population. However, it is crucial to recognize that the success of home-based rehabilitation relies heavily on the adherence of patients to the rehabilitation plan, highlighting the importance of rigorous protocols and regular telemonitoring to ensure patient safety. Further multicenter, long-term randomized controlled trials are warranted to confirm these findings and provide robust evidence to guide the selection of early rehabilitation methods post-knee arthroplasty. Given the evolving landscape of healthcare delivery and the increasing emphasis on patient-centered care, our findings have significant implications for the management of patients after knee arthroplasty.

## Conclusions

Home-based rehabilitation after knee arthroplasty is an effective, safe, and cost-effective alternative to outpatient rehabilitation. These findings, however, need to be confirmed with further multicenter, long-term randomized controlled trials to provide robust evidence to guide the selection of early rehabilitation methods post-knee arthroplasty.

## Data Availability

The datasets used and/or analyzed during the present study are available from the corresponding author on reasonable request.
